# Netrin-1 Improves Functional Recovery through Autophagy Regulation by Activating the AMPK/mTOR Signaling Pathway in Rats with Spinal Cord Injury

**DOI:** 10.1038/srep42288

**Published:** 2017-02-10

**Authors:** Liangjie Bai, Xifan Mei, Zhaoliang Shen, Yunlong Bi, Yajiang Yuan, Zhanpeng Guo, Hongyu Wang, Haosen Zhao, Zipeng Zhou, Chen Wang, Kunming Zhu, Gang Li, Gang Lv

**Affiliations:** 1 Department of Orthopedics, The First Affiliated Hospital of China Medical University, Shenyang, Liaoning, China; 2 Department of Orthopedics, The First Affiliated Hospital of Jinzhou Medical University, Jinzhou Liaoning, China

## Abstract

Autophagy is an process for the degradation of cytoplasmic aggregated proteins and damaged organelles and plays an important role in the development of SCI. In this study, we investigated the therapeutic effect of Netrin-1 and its potential mechanism for autophagy regulation after SCI. A rat model of SCI was established and used for analysis. Results showed that administration of Netrin-1 not only significantly enhanced the phosphorylation of AMP-activated protein kinase (AMPK) but also reduced the phosphorylation of mammalian target of rapamycin (mTOR) and P70S6K. In addition, the expression of Beclin-1 and the ratio of the light-chain 3B-II (LC3B-II)/LC3B-I in the injured spinal cord significantly increased in Netrin-1 group than those in SCI group. Moreover, the ratio of apoptotic neurons in the anterior horn of the spinal cord and the cavity area of spinal cord significantly decreased in Netrin-1 group compared with those in SCI group. In addition, Netrin-1 not only preserved motor neurons but also significantly improved motor fuction of injured rats. These results suggest that Netrin-1 improved functional recovery through autophagy stimulation by activating the AMPK/mTOR signaling pathway in rats with SCI. Thus, Netrin-1 treatment could be a novel therapeutic strategy for SCI.

Spinal cord injury (SCI) is one of the major causes of death and long-term disability among young adults worldwide^1^. Trauma of the spinal cord causes direct mechanical tissue damage (primary injury) and biochemical changes, which induce delayed or progressive cell loss (secondary injury)^2^,^3^. Secondary injuries, including edema, inflammation, excitotoxicity, and axonal disruption, promote neuronal apoptosis, which plays an important role in the physical and functional impairment after SCI^4^. This phenomenon further leads to persistent damage to the tissue around the epicenter of the injury site^5^,^6^. Although the accurate molecular mechanisms of secondary injury remain unclear, blocking or attenuating secondary neuron death may contribute to limit this posttraumatic disabilities^7^,^8^.

Macroautophagy (hereafter called autophagy) is an essential lysosome-dependent cellular catabolic pathway that serves to the degradation of cytoplasmic proteins, protein aggregates, and organelles^9^. Autophagy plays an irreplaceable role in maintaining the balance between the synthesis and degradation of proteins in cells. Previous studies reported that autophagy is important in normal cell growth, differentiation, and survival^10^,^11^. Stimulation of autophagy provides a neuro-protective effect on models of neonatal hypoxia–ischemia-induced brain, closed head, and spinal cord injuries^12^,^13^. However, increased expression of autophagic markers, such as LC3B and autophagosomes, is correlated with enhanced cell death^14^,^15^,^16^. Hence, the role of autophagy remains controversial and a better understanding of the role of autophagy in SCI processes will help us to develop new therapeutic strategies.

Netrins are extracellular, laminin-related proteins that function as guidance cues for cells and axons migrating to their targets during nervous system development. In mammals, the netrin family of proteins are composed of four members, namely, Netrin-1, netrin-2, netrin-3, and β-netrin^17^,^18^. Netrin-1 is expressed in regions of both the developing and adult nervous systems, including optic disk, forebrain, cerebellum, and spinal cord^17^,^19^,^20^,^21^. Netrin-1 is primarily known as a chemotropic factor that attracts or repels axons in the developing nervous system^22^. Moreover, Netrin-1 exerts anti-inflammatory effects by inhibiting leukocyte infiltration in a mouse model of acute pancreatitis^23^; this protein also promotes angiogenesis and decreases the infarct size to enhance recovery after middle cerebral artery occlusion (MCAO) in mice^24^. These properties of Netrin-1in adult animals have aroused our interest to investigate its therapeutic effect and potential mechanism in adult rats after trauma SCI.

The receptor of Netrin-1 is the Down syndrome cell adhesion molecule (DSCAM), which is the gene duplicated in Down syndrome^25^,^26^,^27^,^28^. Netrin-1 could activate AMPK by interacting with DSCAM via the AMPK γ subunit; the activated AMPK may affect actin cytoskeleton by inhibiting mTOR^29^,^30^. The mTOR signaling pathway plays versatile roles in multiple cellular mechanisms, such as cell metabolism, proliferation, and survival^31^. Increasing number of studies reported that stimulation of autophagy by inhibiting the mTOR pathway exhibits neuro-protective effects after SCI. Sekiguchi *et al*.^32^ showed that rapamycin significantly reduced neural tissue damage and promoted locomotor function recovery by stimulation of autophagy via the mTOR pathway after SCI in mouse. Thus, we speculated that Netrin-1 treatment may exert neuro-protective effects through autophagy stimulation by activating the AMPK/mTOR signaling pathway in rats with SCI.

In this study, we aim to investigate the therapeutic effects of Netrin-1 treatment as well as its role on regulation of autophagy and potential mechanisms after SCI. Our findings may provide valuable information to develop therapeutic strategies for SCI. To our best knowledge, this study is the first to investigate the neuro-protective effects of Netrin-1 treatment via autophagy regulation after SCI in rats.

## Results

### Netrin-1 Improves Functional Recovery after SCI

**Figure 1:**
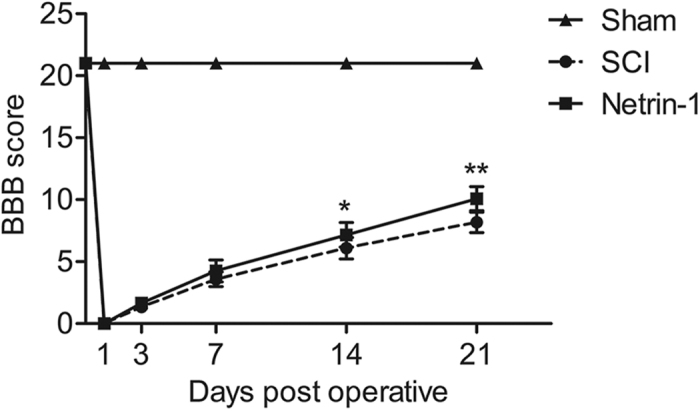
Netrin-1 treatment improves functional recovery after SCI. BBB scores of the sham, SCI, and Netrin-1 groups were evaluated at 0, 1, 3, 7, 14, and 21 days after contusion. *P < 0.05 vs. the SCI group; **P < 0.01 vs. the SCI group. Data represented mean ± SEM (n = 6).

The BBB scores were measured at 0, 1, 3, 7, 14, and 21 days after SCI to evaluate the effect of Netrin-1 treatment on functional recovery. As shown in Fig. 1, the average BBB scores were consistently higher in the Netrin-1 group than those in the SCI group from 3 days to 21 days after contusion. Moreover, the BBB scores of rats in Netrin-1 group and SCI group were same at 0 d and 1d (21 ± 0; 0 ± 0, respectively) after contusion. Althouth, at 3d and 7d after contusion, the BBB scores of rats in Netrin-1 group (3d: 1.67 ± 0.41; 7d: 4.25 ± 0.88) were higher than those in SCI group (3d: 1.33 ± 0.41; 7d: 3.58 ± 0.58), but this did not reach the level of statistical significance. The BBB scores of the Netrin-1-treated rats (7.17 ± 0.98) were significantly higher than those in SCI group (6.08 ± 0.86) at 14 d after contusion (P < 0.05). Netrin-1-treated rats achieved extensive movement of all three joints (hip, knee, and ankle joints) of the hindlimb and some rats occasionally could sweep with no weight support or plantar placement of the paw with no weight support. However. Rats in SCI group could only achieve extensive movement of two joints and slight movement of the third or occasionally appeared extensive movement of all three joints. The BBB scores of rats in Netrin-1 group (10.08 ± 0.97) were also significantly higher than those in SCI group (8.17 ± 0.82) at 21 d after contusion (P < 0.01). Rats in Netrin-1 group could achieved weight supported plantar steps with no forelimb-hindlimb coordination or with forelimb-hindlimb coordination sometimes. However, Rats in SCI group could only sweep with no weight support or plantar placement of the paw with no weight support but none forelimb-hindlimb coordination. In addition, the BBB scores of rats in the sham group were normal and obviously higher than those in the SCI and Netrin-1 groups (P < 0.01, both). These findings indicated that Netrin-1 treatment improves functional recovery after SCI.

### Netrin-1 Reduces the Loss of Motor Neurons and Tissue Damages after SCI

**Figure 2:**
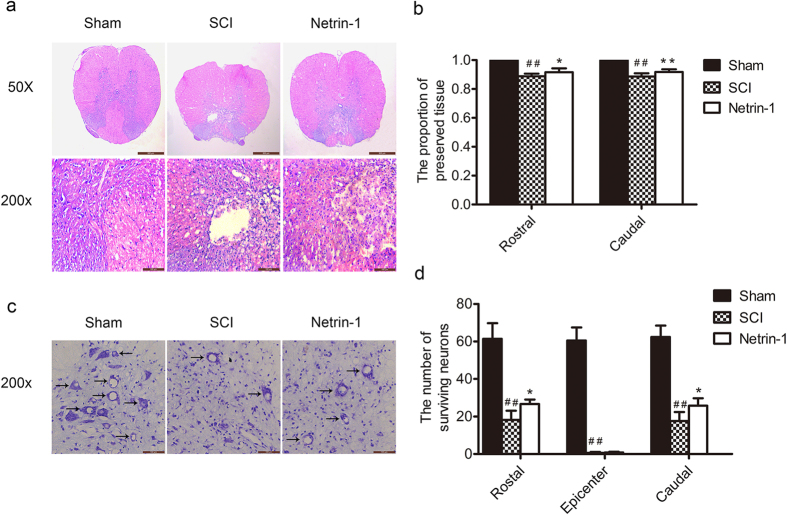
Netrin-1 treatment decreases tissue damage and the loss of neurons after SCI. (**a**) HE staining at 21 days after contusion. Bars = 100 μm (50×) and 100 μm (200×). **(b)** Analysis of the proportion of preserved tissues of the transverse sections at 5 mm rostral and caudal sides; columns represent mean ± SD (n = 5). **(c)** Nissl staining at 21 days after injury. Scale bar = 100 μm (200×). **(d)** Quantification of the number of surviving neurons (black arrows: surviving neurons) at rostral 5 mm, caudal 5 mm, and lesion site. Data represented mean ± SD (n = 5, respectively). In addition, *P < 0.05 vs. the SCI group; **P < 0.01 vs. the SCI group; ^##^P < 0.01 vs. the sham group.

HE staining and Nissl staining were performed 21 days after SCI to evaluate the neuro-protective effect of Netrin-1 treatment. As shown in Fig. 2a, the dorsal white matter and central gray matter exhibited obvious damages in the SCI group relative to that in the sham group. Sections of the SCI group from both 5 mm rostral and caudal sides of injured spinal cord tissue showed a larger cavity involving the dorsal white matter and central gray matter than that from the Netrin-1 group. Quantitative analysis showed that the Netrin-1 group presented a higher proportion of preserved neuron tissues than the SCI group (Fig. 2b). The surviving neurons in the injured spinal cords were counted using Nissl staining (Fig. 2c). Motor neurons of sections at both 5 mm rostral and caudal sides of injured spinal cord tissue were significantly preserved in the anterior horns of the injured spinal cords from the Netrin-1 group compared with those from the SCI group (Fig. 2d). These results suggested that Netrin-1 treatment could reduce the loss of motor neurons and the damages to spinal cord tissues after SCI in rats.

### Netrin-1 Activated AMPK/mTOR Signaling Pathway after SCI

**Figure 3:**
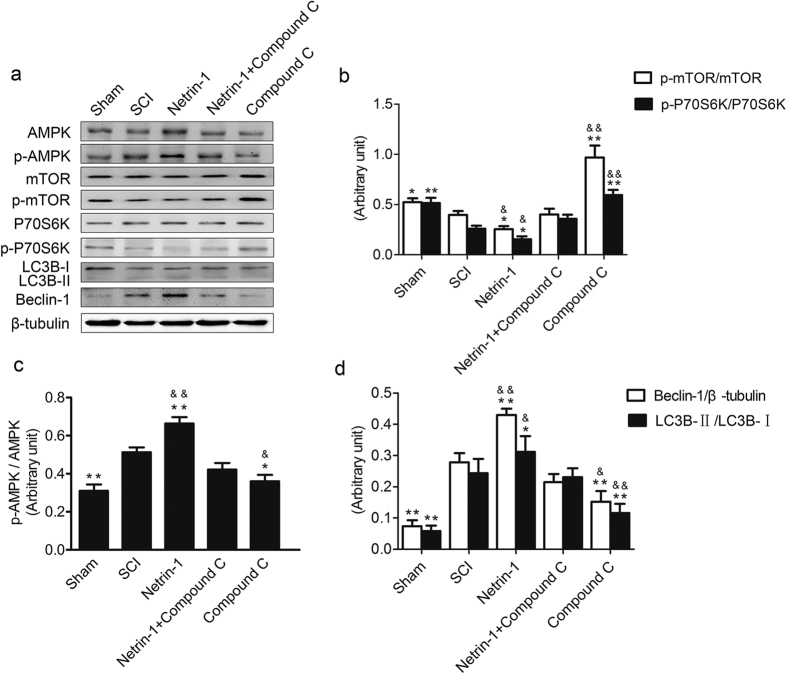
Netrin-1 administration activated the AMPK/mTOR signaling pathway after SCI. **(a)** Expression of AMPK, p-AMPK, mTOR, p-mTOR, p-P70S6K, P70S6K, Beclin-1, LC3B, and β-tubulin in the sham, SCI, Netrin-1, Netrin-1+ compound C, and compound C groups. **(b,c,d)** Quantification analysis of p-mTOR/mTOR p-P70S6K/P70S6K, p-AMPK/AMPK, LC3B-II/LC3B-I, and Beclin-1/β-tubulin in each group. Mean ± SD, n = 3. *P < 0.05 vs. the SCI group; **P < 0.01 vs. the SCI group; ^&^P < 0.05 vs. the Netrin-1+ compound C group; ^&&^P < 0.01 vs. the Netrin-1+ compound C group.

The expression of AMPK, p-AMPK, mTOR, p-mTOR, P70S6K, and p-P70S6K was detected in injured spinal cord tissue by Western blot analysis 3 days after the injury (Fig. 3a–c) to investigate whether the AMPK/mTOR signaling pathway was activated by Netrin-1. mTOR is an important protein in the regulation of autophagy which is negatively regulated by AMPK and results in inhibiting of autophagy. P70S6K, a downstream target protein of mTOR, is positively regulated by mTOR, which represents the activity of the mTOR signaling pathway. AMPK phosphorylation was significantly enhanced and mTOR and P70S6K phosphorylation was significantly reduced in the spinal cord tissues from the SCI group compared with those in the sham group. This condition indicated that the AMPK/m-TOR pathway was activated after SCI. Netrin-1 treatment significantly increased the ratio of p-AMPK/AMPK and significantly decreased the ratio of p-mTOR/mTOR and p-P70S6K/P70S6K. This result implied that Netrin-1 further activated the AMPK/m-TOR signaling pathway after SCI. However, this activation effect was suppressed by compound C. Netrin-1+ compound C group exhibited significantly reduced AMPK phosphorylation and remarkably increased mTOR and P70S6K phosphorylation. Compared with the compound C group, combined application of Netrin-1 and compound C significantly increased ratio of p-AMPK/AMPK and educed the ratio of p-mTOR/mTOR and p-P70S6K/P70S6K; this finding confirmed that Netrin-1 activated the AMPK/mTOR signaling pathway after SCI. Hence, the AMPK/mTOR signaling pathway was activated by Netrin-1 treatment, and this effect was suppressed by compound C.

### Netrin-1 Promotes Autophagy after SCI

**Figure 4:**
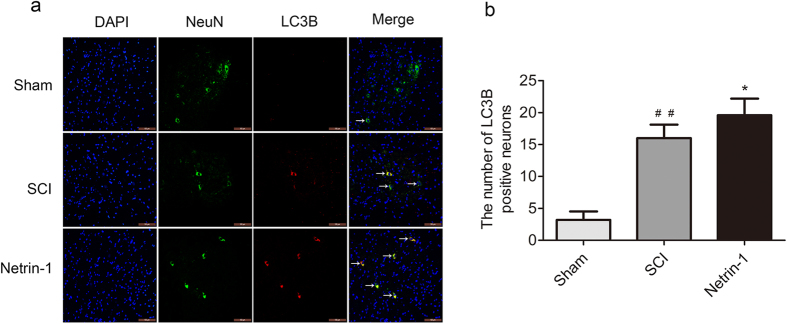
Immunofluorescence analysis of LC3B. **(a)** Double staining for NeuN (green)/LC3B (red) of sections from the spinal cord sample in the sham, SCI, and Netrin-1 groups. Scale bar = 100 μm (white arrows: LC3B-positive neurons). **(b)** Quantification of the number of LC3B-positive neurons in each group. Data were represented as mean ± SD, n = 5. **P < 0.01 vs. the SCI group; ^##^P < 0.01 vs. the sham group.

**Figure 5:**
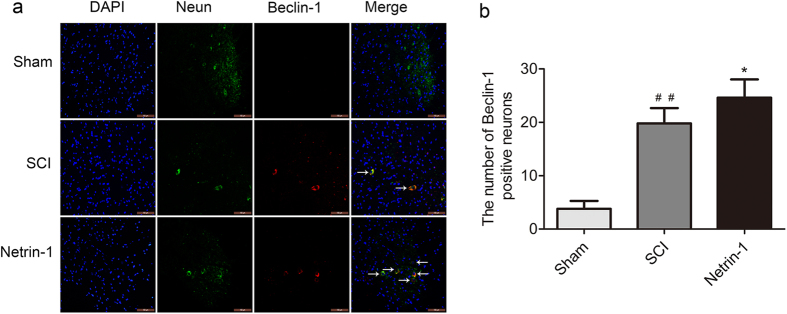
Immunofluorescence analysis of Beclin-1. **(a)** Double staining for NeuN (green)/Beclin-1 (red) of sections from the spinal cord in the sham, SCI, and Netrin-1 groups. Scale bar = 100 μm (white arrows: Beclin-1-positive neurons). **(b)** Quantification of the number of Beclin-1-positive neurons in each group, Data were represented as mean ± SD, n = 5. **P < 0.01 vs. the SCI group; ^##^P < 0.01 vs. the sham group.

The expression of Beclin-1 and LC3B, two accepted biomarkers of autophagy, was detected in injured spinal cord tissue using Western blot analysis 3 days after contusion to investigate the regulatory effect of Netrin-1 on autophagy (Fig. 3a,d). The expression level of Becin-1 and ratio of LC3B-II/LC3B-I significantly increased after SCI compared with that in the sham group. This finding indicated that autophagy was induced after SCI. Moreover, Netrin-1 treatment enhanced the ratio of LC3B-II/LC3B-I and the expression level of Beclin-1 after SCI; hence, autophagy was further induced by Netrin-1 treatment. However, the combined application of Netrin-1 and compound C significantly decreased the expression level of Beclin-1 and the ratio of LC3B-II/LC3B-I compared with those in the Netrin-1 group. This result showed that compound C partly abolished the effect of Netrin-1 on upregulating autophagy. Immunofluorescence staining of LC3B and Beclin-1 was also performed to identify the induction of autophagy at the molecular level. Under increased magnification, double-positive neurons exhibited red punctate LC3B (or Beclin-1) dots in the cytoplasm of green neurons with a blue nucleus (Figs 4a and 5a). The immunofluorescence staining results showed that Beclin-1-positive neurons or LC3B-positive neurons significantly increased in the SCI group compared with that in the sham group. Moreover, the number of Beclin-1-positive neurons or LC3B-positive neurons remarkably increased in the Netrin-1 group compared with that in the SCI group. These findings suggested Netrin-1 treatment could stimulate autophagy after SCI, and this effect was partly abolished by compound C.

### Netrin-1 Inhibits Neuronal Apoptosis After SCI

**Figure 6:**
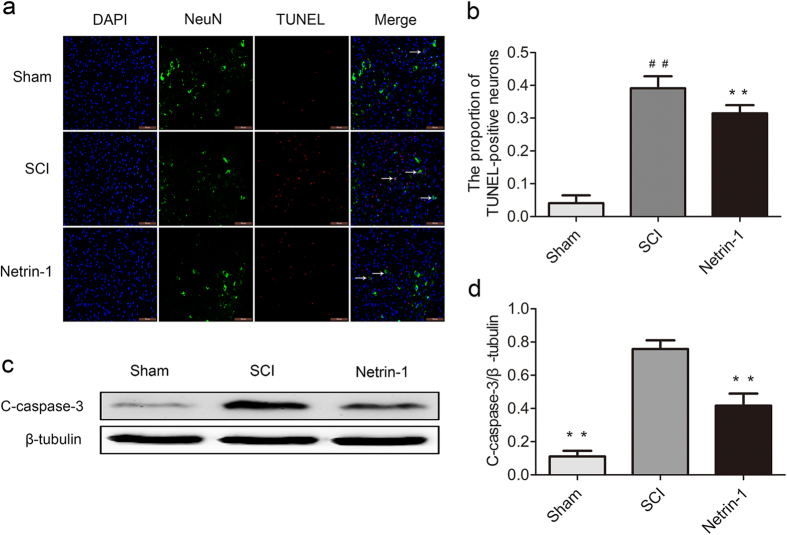
Netrin-1 decreased the number of TUNEL-positive neurons and the expression of C-caspase-3 after SCI. **(a)** TUNEL/NeuN/DAPI double labeling was employed to count the number of TUNEL-positive neurons (white arrows: TUNEL-positive neurons) in the sham, SCI, and Netrin-1 groups. Scale bar = 100 μm. **(b)** Quantification of the ratio of TUNEL-positive neurons in each group. Data were represented as mean ± SD, n = 5. **(c)** Expression of C-caspase-3 in the sham, SCI, and Netrin-1 groups. **(d)** Quantification of the expression of C-caspase-3 in each group. Mean ± SD, n = 3. **P < 0.01 vs. the SCI group; ^##^P < 0.01 vs. the sham group.

The expression of C-caspase-3 in injured spinal cord tissue was detected using Western blot analysis to investigate the anti-apoptosis effect of Netrin-1 after SCI. As shown in Fig. 6c, the expression level of C-caspase-3 significantly increased in the SCI group compared with that in the Sham group (P < 0.01). Moreover, Netrin-1 treatment remarkably reduced the expression level of C-caspase-3 relative to that in the SCI group. To locate the apoptotic neurons, we performed TUNEL/NeuN/DAPI double staining (Fig. 6a). As shown in Fig. 6b, the ratio of TUNEL-positive neurons was significantly higher in the SCI group than that in the Sham group (P < 0.01). Moreover, the ratio of neuronal apoptosis in the Netrin-1 group significantly decreased compared with that in the SCI group (P < 0.01). These findings confirmed that Netrin-1 treatment inhibited neuronal apoptosis after SCI.

## Discussion

In this study, Netrin-1 treatment improved functional recovery and reduced neural tissue damage and loss of neurons by stimulating autophagy via the AMPK/mTOR signaling pathway in rats with SCI. To our best knowledge, this study is the first to reveal the neuro-protective effects of Netrin-1 by stimulation of autophagy after SCI.

SCI is one of the major causes of death and long-term disability among young adults all over the world^33^. The pathology of SCI is usually divided into two phases: primary and secondary injuries. The primary injury is the mechanical impact afflicted directly on the spine; the secondary injury is a complex cascade of molecular events including disturbances in ionic homeostasis, local edema, ischemia, focal hemorrhage, oxidative stress, and inflammatory responses^34^. Although the accurate molecular mechanism of the secondary injury remains unclear, blocking or decreasing secondary neuron death occurring in the secondary injury stage may reduce the disabilities^7^,^8^. Therefore, pharmacological therapeutic strategies for acute SCI should mainly focused on reducing the damages of the secondary injury.

Netrin-1, which belongs to the Netrin family, is not only expressed in the central nervous system during embryonic development but also constitutively expressed by neurons and oligodendrocytes in the spinal cord of adult rats^35^. Netrin-1 is known as a chemotropic factor that either attracts or repels axons, relying on which netrin receptor is expressed in the individual axon^22^. Previous studies mainly investigated the effects of Netrin-1 on the development of nervous system during the embryonic period. Netrin-1 exhibited anti-inflammatory responses in an adult mouse model of acute pancreatitis, thereby promoting angiogenesis and decreasing the infarct size after MCAO in mouse^23^,^24^. Bayat *et al*.^36^ found that Netrin-1 dose-dependently alleviated spatial memory impairment and improved synaptic dysfunction in a rat model of global ischemia. The versatile roles of Netrin-1 in adult animals have gained increased research interest. Thus, we designed this experiment to investigate the therapeutic effects of Netrin-1 treatment and its potential molecular mechanism.

Apoptosis is a process of programmed cell death regulated by multiple signal mechanisms during secondary injury and seriously influences the prognosis after SCI. Activation of C-caspase 3 is a hallmark of apoptotic cell death and functions as the final executor of apoptosis^37^. The present results (Fig. 6c) showed that the expression level of C-caspase 3 in injured spinal cord tissue dramatically increased in the SCI group. Netrin-1 treatment remarkably reduced the activation of C-caspase 3, indicating that the apoptotic signaling pathway was suppressed by Netrin-1. Furthermore, DNA fragmentation was assessed by TUNEL staining. Netrin-1 significantly reduced the ratio of TUNEL-positive neurons in injured spinal cord tissue after SCI (Fig. 6a). These findings suggested that Netrin-1 could inhibit neuronal apoptosis after SCI. In addition, Nissl staining results (Fig. 2c) showed that Netrin-1 administration preserved motor neurons at both 5 mm rostral and caudal sides of injured spinal cord after SCI in rats, indicative of the neuro-protective effect of Netrin-1. HE staining (Fig. 2a) showed that Netrin-1 treatment significantly diminished the area of cavity involving the dorsal white matter and central gray matter at both 5 mm rostral and caudal sides of injured spinal cord after SCI. Moreover, the BBB scores of rats in Netrin-1 group at 14 d (7.17 ± 0.98) and 21d (10.08 ± 0.97) after contusion were significantly higher than those in SCI group at 14 d (6.08 ± 0.86) and 21 d (8.17 ± 0.82) after contusion (P < 0.05; P < 0.01, respectively) (Fig. 1). Hence, Netrin-1 treatment could improve functional recovery in the rat model of SCI. These results suggested the neuro-protective effects of Netrin-1 treatment after SCI. However, the mechanism of neuro-protective effect of Netrin-1 treatment after SCI remains unclear.

Autophagy is an essential lysosome-dependent cellular catabolic pathway involved in the degradation of cytoplasmic proteins, protein aggregates, and organelles. The degradation products can be recycled for synthesis of macromolecules and energy metabolism^38^. Autophagy is an essential process for the maintenance of cellular homeostasis in the central nervous system under normal and stress conditions^39^. Autophagy activity enhanced by different drugs exerts a neuro-protective effect on the central nervous system. For example, rapamycin promotes autophagy and reduces neural tissue damage and locomotor impairment after SCI in mice^32^. Simvastatin improves functional recovery through autophagy induction by inhibiting the mTOR signaling pathway after SCI in rats^40^. Impaired autophagy is involved in neurodegenerative diseases, such as Parkinson’s, Alzheimer’s, and Huntington’s diseases as well as in lysosomal storage disorders^9^,^41^. Beclin-1, a biomarker of autophagy, is involved in the formation of autophagosomes. The conversion of the LC3B-I protein to LC3B-II is another hallmark of autophagy induction during autophagosome formation^16^. In the present study, the Western blots (Fig. 2a,d) showed that the expression level of Beclin-1 and the ratio of LC3B-II/LC3B-I in injured spinal cord tissue significantly increased after SCI. Immunofluorescence analysis (Figs 3 and 4) indicated that the number of Beclin-1-positive neurons or LC3B-positive neurons in injured spinal cord tissue remarkably increased after SCI. Similarly, previous studies reported that autophagy activity was induced after SCI^32^. These findings indicated that Netrin-1 could stimulate autophagy activity after SCI in rats. We speculate that the neuro-protective effect of Netrin-1 may be associated with upregulated autophagy activity after SCI. However, some studies showed that autophagy may promote cell death through excessive self-digestion, degradation of essential cellular constituents, and/or interaction with apoptotic cascades^42^. Kanno *et al*.^14^ found that the increased expression of Beclin-1 activates autophagy and induced autophagic cell death at the lesion site after SCI. Although autophagy-induced apoptosis is one of the major events in the secondary injury in SCI, the potential underlying mechanism remains unclear^43^. Therefore, the protective or harmful effects of autophagy activity on neuronal tissues after SCI remain controversial. Basing on the paradoxical role of autophagy after SCI, we tentatively suggest that different injured models, injury degrees, and administration times may differentially affect autophagy activation, leading to varied outcomes. Hence, in-depth studies must be conducted to clarify the relationship between autophagy and apoptosis as well as its underlying molecular mechanism after SCI.

AMPK is a serine/threonine protein kinase that consists of three subunits: a catalytic α-subunit and regulatory β- and γ-subunits^44^,^45^,^46^. AMPK is not only an important metabolic sensor of energy balance at the cell and organism levels^47^ but also plays an important role in neuronal development by modulating neurite outgrowth^29^,^48^. Activated AMPK (p-AMPK) leads to phosphorylation and activation of the TSC1/2 complex, which inhibits mTOR activity^49^. mTOR is a serine/threonine protein kinase that regulates cell metabolism, proliferation, death, and survival; this protein also participates in some physiological processes, including transcription, mRNA turnover and translation, ribosomal biogenesis, vesicular trafficking, autophagy, and cytoskeletal organization^50^. Previous studies showed that the inhibition of the mTOR signaling pathway exerted neuro-protective effects by stimulating autophagy in the central nervous system. For example, rapamycin reduced neural tissue damage after SCI in mice through autophagy stimulation by inhibiting the mTOR signaling pathway^32^. Moreover, metformin could promote autophagy via the AMPK/mTOR signaling pathway to improve prognosis after SCI^51^. AMPK interacts with DSCAM, a Netrin-1 receptor, and plays an important role in Netrin-1 induced neurite outgrowth in cortical neurons^30^. Inhibition of the AMPK/mTOR signaling pathway could stimulate autophagy exerting neuro-protective effects after SCI. We investigated whether or not Netrin-1 exhibited the same functions. In the current study, the Western blot (Fig. 2a–c) showed that Netrin-1 treatment activated the AMPK/mTOR signaling pathway in injured spinal cord tissue and this effect could be partly suppressed by compound C. Similarly, previous research reported that Netrin-1 could inhibit mTOR by activating AMPK^30^. Basing on Western blot and immunofluorescence staining results for Beclin-1 and LC3B (Figs 4 and 5), we demonstrated that Netrin-1 could stimulate autophagy in injured spinal cord tissue after SCI in rats. The effect of Netrin-1 on regulating autophagy was partly abolished by compound C. These data may suggest that Netrin-1 stimulated autophagy by activating the AMPK/mTOR signaling pathway. Compared with compound C alone, the combined application of Netrin-1 and compound C could significantly increase the ratio of p-AMPK/AMPK, decrease ratios of p-mTOR/mTOR and p-P70S6K/P70S6K, and enhance the expression of Beclin-1 and ratio of LC3B-II/LC3B-I. This result confirmed that Netrin-1 stimulated autophagy by activating the AMPK/mTOR signaling pathway after SCI. However, we cannot rule out that Netrin-1 could stimulate autophagy through other potential signaling pathways after SCI. Hence, in-depth studies need to be conducted to investigate other potential molecular mechanisms of Netrin-1 in autophagy stimulation after SCI.

This work presents several limitations. First, further related research should be conducted, such as in primary neurons, because of lacking evidence from *in vitro* experiments. Second, further studies must be conducted to investigate other potential effects of Netrin-1, such as anti-inflammatory response and promotion of angiogenesis after SCI as well as their underlying mechanisms.

In summary, our results showed that Netrin-1 treatment exerts neuro-protective effect through autophagy stimulation by activating the AMPK/mTOR signaling pathway after SCI in rats. Hence, Netrin-1 may provide potential therapeutic strategies to improve functional recovery after SCI.

## Materials and Methods

### Reagents and Antibodies

Recombinant rat Netrin-1 was purchased from Creative Biomart (Shirley, NY, USA). Anti-LC3B, anti-Beclin-1, anti-NeuN, anti-mTOR, and anti-p-mTOR antibody as well as goat anti-rabbit and goat anti-mouse-IgG HRP were purchased from Abcam (Cambridge, MA, USA). Anti-AMPK, anti-p-AMPK, anti-p-P70S6K, anti-P70S6K, and anti-caspase-3 antibody were obtained from Cell Signaling Technology (Danvers, MA, USA). Alexa Fluor^®^ 568 and Alexa Fluor^®^ 488 were acquired from Life Technology (Carlsbad, CA, USA). Anti-β-tubulin antibody was provided by TransGen Biotech (Beijing, China). Dorsomorphin (Compound C), an AMPK inhibitor, was supplied by Selleck Chemicals LLC (Houston, TX, USA). An enhanced chemiluminescence (ECL) kit was obtained from Beyotime Institute of Biotechnology (Nanjing, Jiangsu, China). *In situ* Cell Death Detection Kit was purchased from Roche (Mannheim, Germany). All of the other reagents were acquired from Sigma–Aldrich (St. Louis, MO, USA) unless otherwise specified.

### Animals and Model of SCI

All procedures were approved by the ethics committee of China Medical University. Adult female Sprague–Dawley rats (220–250 g) were purchased from the Animal Laboratory of China Medical University in Shenyang, China. This research was approved by the Committee of Ethics on Animal Experiment of China Medical University. Experiments were carried out under the control of the Guidelines for Animal Experiments. The rats were housed under standard temperature conditions with controlled 12-h light/dark cycles and given free access to food and water. A rat model of SCI was established using the modified weight-drop method as previously described^52^. The rats were injected with 10% chloral hydrate (3.0 ml/kg, i.p.) anesthesia and positioned on a cork platform. The skin was incised along the midline of the dorsum to expose the vertebral column and perform laminectomy at T9 level. A 10-g impactor device (diameter: 2 mm) made by ourselves was subsequently dropped from a height of 25 mm to the spinal cord and removed immediately without disrupting the intact of dura. This condition resulted in flicking of the hind legs, appearance of tail sway reflex, and lower limb paralysis. After rinsing with sterile saline, the muscles and skin were sutured in layers. After the surgery, all rats, except those in the sham group underwent manual urinary bladder emptying three times daily until thshed.

### Experimental Groups and Drug Treatments

Rats were randomly divided into five groups after adapting to the new environment. Recombinant rat Netrin-1 was dissolved in 1× phosphate buffered saline (PBS) to achieve a final concentration of 800 ng/ml. Compound C was dissolved in 1× PBS and given at a dose of 20 mg/kg. The administration dose of Netrin-1 and compound C was selected based on previous studies^36^,^53^.

1. Sham group: Rats were subjected to laminectomy and immediately treated with 1× PBS (1 ml) by intraperitoneal injection (i.p.) once daily for the next 2 days (n = 30).
2. SCI group: After inducing SCI, the rats were immediately administrated with 1 ml of 1× PBS (i.p.) once daily for the next 2 days (n = 30).
3. Netrin-1 group: After SCI, the rats were immediately administrated with Netrin-1 (800 ng/ml, 1 ml) (i.p.) once daily for the next 2 days (n = 30).
4. Netrin-1+ compound C group: After contusion, the rats were immediately injected with Netrin-1 (800 ng/ml, 1 ml) and Compound C (20 mg/kg) once daily for the next 2 days (n = 6).
5. Compound C group: After SCI, rats were immediately administrated with compound C (20 mg/kg) and 1× PBS (1 ml) once daily for the next 2 days (n = 6).

### Locomotion Recovery Assessment

The Basso, Beattie, and Bresnahan (BBB) locomotor rating scale^54^ was used to evaluate the recovery of behavioral function at 0, 1, 3, 7, 14, and 21 days after contusion. The BBB scores range from 0 (complete paralysis) to 21 (normal locomotion). Before sacrificing, rats were placed individually in a molded plastic open field for 4 min to ensure that the maximum scores are obtained. Rats from each group (n = 6) were simultaneously evaluated by two trained observers who were blinded to the study. The means of scores were used for analysis.

### Tissue Preparation

At 3 and 21 days after SCI, the rats were overdosed with sodium pentobarbital (100 mg/kg) via i.p. The rats were transcardially perfused with 0.9% saline, followed by 4% paraformaldehyde in 0.1 M 1× PBS (pH = 7.4). The spinal cord segments (2 cm rostral and caudal to the epicenter) were fixed in 4% paraformaldehyde overnight. The segments were then transferred into 30% sucrose in 4% paraformaldehyde until reaching the bottom. Serial 10 μm transverse frozen sections were cut with a cryostat at −20 °C.

### Hematoxylin–Eosin (HE) Staining and Histological Analysis

HE staining was performed using the sections obtained 21 days after SCI. Two sections from each rat were selected at 5 mm rostral and caudal to the epicenter for histopathological examination by HE staining (n = 5 per group). The sections were hydrated using a series of ethanol and then stained with HE solution in accordance with the manufacturer’s instructions. The cavity area on each section was measured using IMAGE J software. The measurements were performed in accordance with the methods presented in a previous study^55^.

### Nissl Staining and Cell Counting of Ventral Motor Neurons

The sections obtained 21 days after SCI were used for Nissl staining. Three sections were randomly selected at the lesion site and at the 5 mm rostral and caudal sides from each rat (n = 5 per group). The sections were incubated in 0.1% Cresyl violet Nissl staining solution in accordance with the manufacturer’s instructions. The quantity of ventral motor neurons of three sections from each rat were respectively counted under a light microscope at high magnification. Then the average quantities of ventral motor neurons from the lesion site and at the 5 mm rostral and caudal sides were respectively calculated and used for analysis (scale bar = 200).

### Western Blot analysis

Rats were given with 10% chloral hydrate (3.0 ml/kg, i.p.) anesthesia. The spinal cord tissues (0.5 cm length around the epicenter) of the rats were removed rapidly and stored at −80 °C for Western blot analysis 3 days after SCI. The samples were homogenized in RIPA buffer containing PMSF for 30 min on ice. The debris was removed after the complex was centrifuged at 12,000 rpm (30 min, 4 °C). The supernatant was obtained for protein assay. After determining the protein levels in the lysates with BCA Protein Assay Kit, 40 μg of the protein was separated using sodium dodecyl sulfate–polyacrylamide gel electrophoresis and transferred onto a polyvinylidene difluoride membrane. The membrane was blocked with 1% bovine serum albumin in TBS with 0.1% Tween-20 for 2 h at room temperature and incubated at 4 °C overnight with the following primary antibody solutions: anti-LC3B antibody (1:1000), anti-Beclin-1 antibody (1:1000), anti-AMPK antibody (1:1000), anti-p-AMPK antibody (1:2000), anti-mTOR antibody (1:1000), anti-p-mTOR antibody (1:2000), anti-P70S6K antibody (1:1000), anti-p-P70S6K antibody (1:1000), anti-Caspase-3 antibody (1:1000), and anti-β-tubulin antibody (1:2000). The membranes were then incubated with the corresponding secondary antibodies for 2 h at room temperature. The immunoreactive bands were developed using ChemiDoc-It^TM^TS2 Imager (UVP, LLC, Upland, CA, USA) and analyzed using ImageJ2× software program (National Institute of Health, Bethesda, MD, USA). The experiments were repeated three times.

### Immunofluorescence Staining

The sections obtained 3 days after SCI were used for immunofluorescence staining. Three sections were randomly selected at the lesion site and at 5 mm rostral and caudal sides to the epicenter from each rat (n = 5 per group) for immunofluorescence staining. The transverse 10 μm sections were incubated with 5% normal goat serum in 1× PBS containing 0.1% Triton X-100 for 2 h at 4 °C. The sections were then incubated with primary antibody, namely, anti-NeuN antibody (1:500) at 4 °C overnight. The sections were rinsed three times with 1× PBS and incubated with Alexa Fluor^®^ 488 (1:400) for 2 h at room temperature. Subsequently, the sections were incubated with the corresponding secondary antibodies, including anti-LC3B antibody (1:200) and anti-Beclin-1 antibody (1:100), for 2 h at room temperature. After rinsing three times with 1× PBS, the sections were incubated with Alexa Fluor^®^ 568 for 2 h at room temperature. The sections were rinsed three times with 1× PBS and incubated with 4,6-diamidino-2-phenylindole (DAPI) for 15 min. Finally, the sections were rinsed three times with 1 × PBS and sealed with a coverslip. All images were captured with a fluorescence microscope (Leica, Heidelberg, Germany). The number of double-labeled positive neurons of LC3B or Beclin-1 were counted at 10 randomly chosen fields within each section and sum up. The total number of positive neurons of three sections from each rat were calculated and used for analysis.

### Transferase UTP Nick End Labeling (TUNEL) Staining

The sections obtained 3 days after SCI were used for TUNEL staining. Three sections were randomly selected at the lesion site and at 5 mm rostral and caudal sides to the epicenter from each rat (n = 5 per group). The sections were incubated with 5% normal goat serum in 1× PBS containing 0.1% Triton X-100 for 2 h at 4 °C and further incubated with anti-NeuN antibody (1:500) overnight at 4 °C. Subsequently, the sections were incubated with Alexa Fluor^®^ 488 (1:400) for 2 h at room temperature after rinsing three times with 1× PBS. The rinsed sections were incubated with TUNEL staining solution from the *In situ* Cell Death Detection Kit in accordance with the manufacturer’s instructions. After rinsing three times with1× PBS, the sections were incubated with DAPI for 15 min prior to capturing images by using a fluorescence microscope (Leica, Heidelberg, Germany). Red TUNEL dots located in green neurons with a blue nucleus were identified as apoptotic neurons. The total number of neurons and apoptotic neurons of three sections from each rat were counted and then ratios were calculated.

### Statistical Analysis

Data were presented as mean ± standard deviation (SD) and analyzed by SPSS 19.0. Comparisons between two groups and among multiple groups were performed using unpaired Student’s t-test and one-way ANOVA, respectively. The significance of the BBB scores was analyzed by Mann–Whitney’s U test. P values < 0.05 were considered statistically significant.

## Additional Information

**How to cite this article:** Bai, L. *et al*. Netrin-1 Improves Functional Recovery through Autophagy Regulation by Activating the AMPK/mTOR Signaling Pathway in Rats with Spinal Cord Injury. *Sci. Rep.* **7**, 42288; doi: 10.1038/srep42288 (2017).

**Publisher's note:** Springer Nature remains neutral with regard to jurisdictional claims in published maps and institutional affiliations.
